# Medication therapy patients with juvenile idiopathic arthritis (JIA), who needed joint replacement in adult life

**DOI:** 10.1186/1546-0096-12-S1-P154

**Published:** 2014-09-17

**Authors:** Tatiana A Shelepina, Natalia Stepanenko, Dmitriy Ivanov

**Affiliations:** 1 Pediatric Rheumatology, Scientific Research Institute Of Rheumatology, Moscow, Russian Federation; 2 Orthopedics, Scientific Research Institute Of Rheumatology, Moscow, Russian Federation

## Introduction

Well-timed administration of disease-modifying therapy is a keystone of contemporary treatment for patients with JIA.

## Objectives

The objective is to analyze the effect of medication therapy carried out for patients in the first year of the disease and timeframe for disease-modifying therapy administration.

## Methods

23 adult patients of orthopedic surgery department who underwent joint replacement in 2010–2013. Based on patients survey and medical documents, we analyzed the age of the disease onset, arthritis type, mode of therapy taken in the first year of the disease, duration of the disease by the time of disease-modifying therapy administration and social status during the study (education, job, marital status, age and disease duration by the time of the joint replacement).

## Results

87% of the patients examined were women. Mean age of the disease onset is 4.6 years (2–15 years). Polyarticular type was found in 13 patients (56%), systemic and oligoarticular types were observed in 22% each. In the first year of the disease 4 patients were treated with glucocorticoids (GC) (intra-articular and systemic injection); non-steroid anti-inflammatory drugs (NSAID) were administered in 8 patients; 8 patients received combined therapy with these drugs and one woman received disease-modifying therapy in addition to the combination of these drugs. Average duration of the disease by the time of disease-modifying therapy administration was 6.5 years. In fact, 3 patients received methotrexate starting from the disease onset, two patients were treated only in hospital conditions. All patients finished school, 8 patients have been educated at home: a girl since the second form and others in senior school. 6 patients had vocational secondary education, 7 patients had a higher education, 7 patients were employed, 3 patients went on to further study, 5 patients had a family 3 of which were mothers. Mean age of the patients by the time of the first joint replacement was 23 years (17–35 years) and average disease duration was 15.9 years. One joint was replaced in 70% of patients, two joints in 20% and three joints in 10%. 60% of patients underwent hip replacement and 40% underwent knee replacement.

## Conclusion

This treatment was inadequate in the majority of patients, though the condition of some patients was relatively satisfactory for a long period that allowed them to attend school. In 60% of patients with early onset of the disease, 47 % had impairment in adolescent period.In one woman elbow replacement was associated with an error in rehabilitation treatment in childhood: redressment after short duration of the disease led to fast ankylosing. Severe functional disorders and low social activity were caused by inadequate therapy administered at the early stage of the disease. Nowadays, almost all patients with chronic arthritis receive disease-modifying therapy in the first 3–6 months, which inspires hope for better functional and social results of their treatment in adult life.

## Disclosure of interest

None declared.

